# Concomitant Respiratory Failure Can Impair Myocardial Oxygenation in Patients with Acute Cardiogenic Shock Supported by VA-ECMO

**DOI:** 10.1007/s12265-021-10110-2

**Published:** 2021-02-23

**Authors:** Anthony R. Prisco, Jazmin Aguado-Sierra, Constantine Butakoff, Mariano Vazquez, Guillaume Houzeaux, Beatriz Eguzkitza, Jason A. Bartos, Demetris Yannopoulos, Ganesh Raveendran, Mikayle Holm, Tinen Iles, Claudius Mahr, Paul A. Iaizzo

**Affiliations:** 1 Department of Medicine, Division of Cardiology, University of Minnesota, Minneapolis, MN USA; 2 Barcelona Supercomputing Center – Centro Nacional de Supercomputación, Barcelona, Spain; 3 ELEM Biotech, Barcelona, Spain; 4 Department of Biomedical Engineering, University of Minnesota, Minneapolis, MN USA; 5 Department of Surgery, Visible Heart® Laboratories, University of Minnesota Medical School, B172 Mayo, MMC 195, 420 Delaware Street SE, Minneapolis, MN 55455 USA; 6 Department of Medicine, Division of Cardiology, University of Washington, Seattle, WA USA

**Keywords:** VA-ECMO, Acute respiratory distress syndrome, North-south syndrome, Computational fluid dynamics

## Abstract

Venous-arterial extracorporeal membrane oxygenation (VA-ECMO) treatment for acute cardiogenic shock in patients who also have acute lung injury predisposes development of a serious complication called “north-south syndrome” (NSS) which causes cerebral hypoxia. NSS is poorly characterized and hemodynamic studies have focused on cerebral perfusion ignoring the heart. We hypothesized in NSS the heart would be more likely to receive hypoxemic blood than the brain due to the proximity of the coronary arteries to the aortic annulus. To test this, we conducted a computational fluid dynamics simulation of blood flow in a human supported by VA-ECMO. Simulations quantified the fraction of blood at each aortic branching vessel originating from residual native cardiac output versus VA-ECMO. As residual cardiac function was increased, simulations demonstrated myocardial hypoxia would develop prior to cerebral hypoxia. These results illustrate the conditions where NSS will develop and the relative cardiac function that will lead to organ-specific hypoxia.

**Graphical Abstract:**

**Figure:**
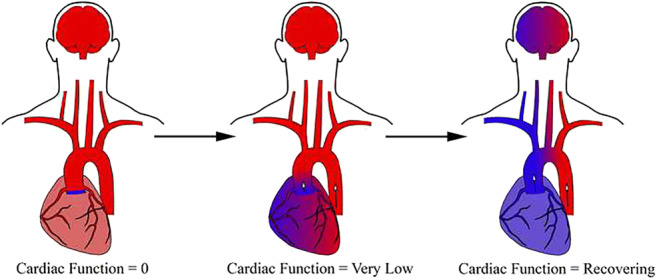
Illustration of the impact of north-south syndrome on organ-specific oxygen delivery. Patients on VA-ECMO have two sources of blood flow, one from the VA-ECMO circuit and one from the residual cardiac function. When there is no residual cardiac function, all organs are perfused with oxygenated blood. As myocardial recovery progresses, blood supply from the two sources will begin to mix resulting in non-homogeneous mixing and differential oxygenation based upon the anatomical site of branching vessels.

**Supplementary Information:**

The online version contains supplementary material available at 10.1007/s12265-021-10110-2.

## Introduction

Acute heart failure refractory to inotrope therapy is usually treated with mechanical circulatory support (MCS) devices [1]. There are several MCS devices to treat acute heart failure, one of the most common being venous-arterial extracorporeal membrane oxygenation (VA-ECMO). VA-ECMO is advantageous as it can provide biventricular support and external oxygenation [2]. Peripheral VA-ECMO is typically configured where blood is removed from the patient’s inferior vena cava, externally oxygenated, and then pumped back into the patient’s body via the femoral artery. This configuration results in oxygenated blood flowing retrograde through the aorta [3]. In this scenario, arterial blood flow comes from two sources: the VA-ECMO MCS device, and the patient’s residual native cardiac function.

The antegrade blood flow from native cardiac function and the retrograde blood flow from the VA-ECMO device are oxygenated independently each perfusing separate areas of the body. Therefore, both sources must be well oxygenated or areas of the body may become locally hypoxic. Blood flow from residual cardiac function relies on the patient’s intrinsic lung function for oxygenation while the VA-ECMO circuit relies on an external oxygenator [4]. The site in the aorta at which these two jets mix is directly related to the pressure and volumetric flow of both jets [5]. Depending upon the underlying disease process, residual cardiac function can potentially represent a substantial portion of the total blood flow through a patient [4].

Mixing of the two jets does not cause significant hypoxia-related complications unless there is concomitant lung injury, often acute respiratory distress syndrome (ARDS) [6]. A patient with ARDS and residual cardiac function will pump hypoxemic blood antegrade into the aorta as it does not pass through the external oxygenator. This can become clinically significant as blood within the large arteries will not be uniformly oxygenated as the complex hemodynamics of the two jets prevents homogeneous mixing [3]. The majority of blood proximal to the mixing site of the two streams is hypoxemic. This causes a rare phenomenon called “north-south syndrome” (NSS), otherwise known as “dual circulation” [7]. NSS is rapidly fatal when hypoxia reaches the vessels supplying the brain.

NSS is most commonly observed in patients who have underlying cardiogenic shock secondary to a cardiomyopathy induced from a primary lung process (bacterial or viral pneumonia). Additionally, NSS can also develop in patients who require VA-ECMO as a treatment for a primary cardiac arrest (i.e., STEMI resulting in cardiogenic shock). In these patients, respiratory failure is typically the result of resuscitation or prolonged ICU cares (i.e., development of ARDS due to aspiration or ventilator-associated pneumonia). Early recognition of NSS is crucial as treatment requires reconfiguring VA-ECMO to VAV-ECMO or switching the patient to VV-ECMO if there is adequate cardiac function [3].

NSS is clinically important because once developed, there is potential for the entire blood supply to the brain to become hypoxemic [8]. To monitor for cerebral hypoxia in the setting of NSS, arterial blood is sampled or pulse oximetry is placed on the patient’s right hand [9]. This is because the blood supply from the right arm comes from the innominate artery and is the most proximal branch point off the aorta, aside from the coronary arteries. Therefore, the right hand represents the worst-case scenario of cerebral oxygenation for patients on VA-ECMO. Prior studies have focused on the management of NSS with attention towards avoiding cerebral hypoxia. Unfortunately, no studies have investigated myocardial oxygenation.

Myocardial oxygen delivery is important because hypoxemic blood supplied to the heart in a critically ill patient in cardiogenic shock would likely induce further injury via ischemia ultimately impairing myocardial recovery. This has not been reported in the literature as localized myocardial oxygenation in patients on VA-ECMO is not monitored as it is difficult to measure clinically. In absence of clinical measurement of local myocardial oxygenation, impaired myocardial recovery secondary to NSS-induced ischemia would be most likely attributed to the primary cause of the acute cardiogenic shock rather than impaired recovery due to NSS-induced ischemia. Finally, many studies have discussed NSS and shown intuitive illustrations of the phenomena; however, there are a paucity of studies that investigated and quantified the complex hemodynamics occurring in a patient supported by VA-ECMO.

For the present study, we created a 3D mathematical model of the aorta and large branching vessels from a human cadaver. An ECMO cannula was placed in the right iliac artery. We then conducted clinically realistic blood flow simulations representing a patient who was being supported by VA-ECMO. Simulations were done at varying levels of residual cardiac function. To determine how hypoxemic blood from residual cardiac function might affect end-organ oxygenation (focused on the brain and heart), we tracked blood flow from the two sources (residual cardiac output and VA-ECMO) and quantified the relative amounts of blood flow from each source at each major aortic branching vessel.

## Methods

### Creation of Patient Model

**Fig. 1:**
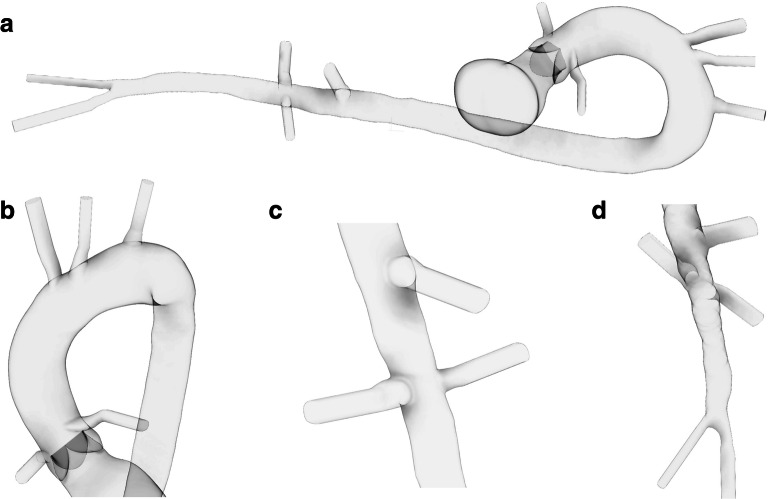
Geometric creation of aorta and branching vessels. The left ventricle, aorta, and major branching vessels from a whole-body CT scan of a cadaver were manually segmented and recreated (**a**). LV was removed and replaced with a rigid tube for simulations. Major vessels included the coronary, innominate, left carotid, left subclavian (**b**), superior mesenteric, renal (**c**), and iliac (**d**). Extruded extensions were placed on the ends of all vessels to stabilize simulations

A 3D model of the human aorta and branching vessels was created from a de-identified contrast-enhanced whole-body computed tomography (CT) scan of a human cadaver. The cadaver was obtained through the University of Minnesota’s Bequest Program in a partnership with the Department of Surgery’s Visible Heart® Laboratories (VHL) (http://www.vhlab.umn.edu/atlas/histories/dicomrequest.shtml). The cadaver was a 68.0 kg male who was 178 cm tall and had no significant medical history prior to death. The blood flow path within the aorta and branching vessels was manually segmented in Mimics v20.0 (Materialise Inc., Leuven, Belgium) and converted to a 3D object using 3-Matic v13.0 (Materialise Inc., Leuven, Belgium). Extruded extensions were created at each vessel boundary to stabilize simulations. The vasculature reconstructed in the 3D model included the entire aorta and proximal portion of the following arteries: left and right coronaries, innominate, left carotid, left subclavian, superior mesenteric, left and right renal, and left and right iliac. An idealized aortic valve was placed in the proximal aorta, and a VA-ECMO cannula was placed in the patient’s right iliac artery (Fig 1).

### CFD Simulations

The geometry was reconstructed into a rigid mesh of 4.8 million elements using ANSA v17.0 (Beta Simulation Solutions, Lucerne, Switzerland). Transient 3D CFD simulations were conducted using Alya, a high-performance finite-element multiphysics simulation software (Barcelona Supercomputing Center, Barcelona, Spain) at a time step of 0.0005 s [10]. Simulations were solved on the Joliot-Curie Rome Supercomputer (CEA/GENCI, France) with computing time awarded through the PRACE COVID-19 Fast Track Call. Simulations were run for a total of (20) 0.75-s cardiac cycles, which corresponds to a heart rate of 80 bpm. This amount of simulation time was chosen as this was the amount of time required to achieve steady behavior at each outlet. The simulation boundary conditions were modeled as follows:

1. A 15 Fr ECMO cannula was virtually placed at the distal end of the right iliac artery and continuous retrograde flow was modeled at 4.0 L/min through the ECMO cannula into the right iliac artery. Prior to exiting the ECMO cannula, flow was fully developed.
2. The independent variable, residual cardiac output, was modeled from the ascending aortic waveform of volumetric blood flow measured by 4D MRI by Burke et al. [11]. The waveform was then scaled to cardiac indexes (cardiac index = cardiac output/body surface area) of 0, 0.5, 1.0, 1.5, and 2.0 (L/min·m^2^). Body surface area for the cadaver was calculated from the Dubois equation (BSA = 0.007184 W^0.425^H^0.725^) [12]. Additional simulations were completed where the cardiac index was held constant at 1.0 L/min·m^2^ and flow from VA-ECMO was varied from 2.0 to 6.0 L/min.
3. Outlets included were right/left coronary, innominate, left carotid, left subclavian, superior mesenteric, right/left renal, and left iliac arteries. All boundary conditions for each of these vessels were 3 element Windkessel models parameterized to physiologically realistic flows consistent with previous studies [13–15]. Further details are in the supplemental information.
4. All walls were approximated as no-slip, and blood was modeled as a Newtonian fluid with a viscosity of 0.0035 Pa·s and density of 1060 kg/m^3^, equivalent to that of blood at 37°C, consistent with previous studies [16, 17]. A total of 250 randomly distributed particles with size/density equal to that of platelets were injected into each inlet (Proximal Aorta, VA-ECMO Inflow Cannula) every 20 ms. Particles were labeled according to the respective inlet and followed using Lagrangian particle tracking [18], and the number exiting each outlet was quantified after the beat to beta quantities stabilized. The approximate distribution of particles at each vessel outlet provided the blood flow origin quantification. From these data, flow measurements were determined and plotted (Figs. 3 and 4).

### Patient Selection

Retrospective review of all patients who underwent VA-ECMO for acute cardiogenic shock (between 12/2015 and 12/2019) was approved by the institutional review board at the University of Minnesota (No. 170M11301). Patients were then identified who had concomitant respiratory failure and acute cardiogenic shock. After reviewing those patients, those with regular serum cardiac-specific troponin measurements (biomarker of myocardial necrosis) were identified and troponin concentrations were plotted as a function of time (Fig. 6).

## Results

### Site of Mixing

VA-ECMO flow was simulated at 4.0 L/min retrograde through the right iliac artery. The independent variable, residual cardiac output, was simulated in an incrementally increasing fashion starting at a cardiac index of 0.0 L/min·m^2^ and progressing up to 2.0 L/min·m^2^ at increments of 0.5 L/min·m^2^. Blood was then tracked spatially from its origin (i.e., from the patient’s heart or from the VA-ECMO circuit) by injecting labeled particles at each source. Particles from the patient’s heart were colored blue, while particles from the VA-ECMO circuit were colored red. It should be noted that relative oxygenation was not quantified in these simulations as the degree of oxygen saturation could vary at both sources depending upon the clinical situation. Assignment of oxygen saturation would be arbitrary. Instead, residual cardiac output (cardiac output of acutely failing heart) was colored blue to demonstrate relative hypoxemia as compared to blood flow from the VA-ECMO circuit, which was colored red.

**Fig. 2:**
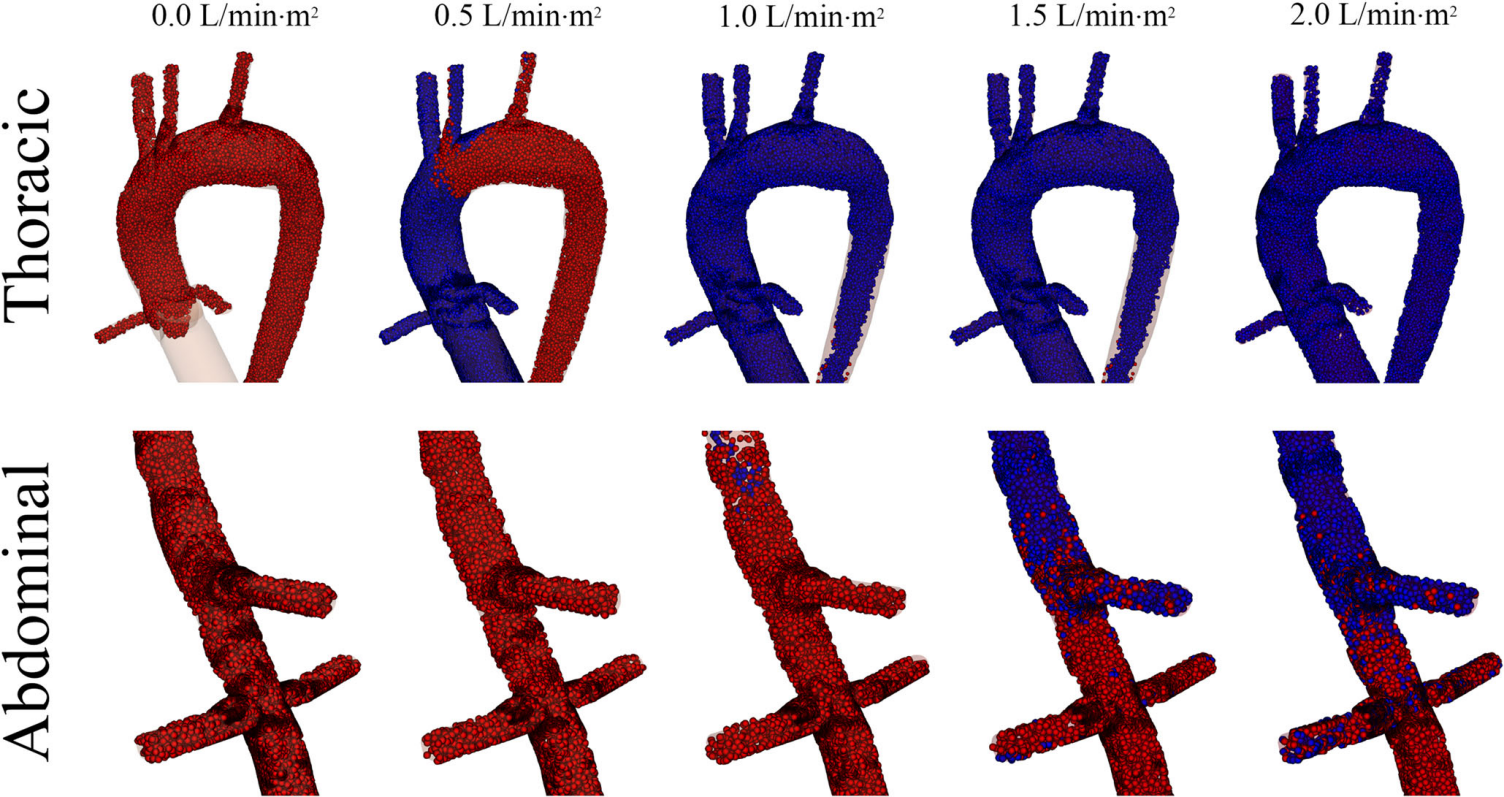
Mixing site as function of residual cardiac function. The site at which the two sources of blood mix was visualized after the simulations reached steady state. As the residual cardiac function increased, the site of mixing shifted distally, starting in the thoracic aorta (residual index of 0.5 L/min·m^2^) reaching the abdominal aorta (residual index of 2.0 L/min·m^2^)


Particles were injected at each respective inlet and the simulations were completed for 20 cardiac cycles. The site of mixing is shown in Fig. 2. The distance from the aortic annulus at which the jets of blood mixed was directly proportional to the residual cardiac output. Figure 2 and Video 1 demonstrate that the predominant source of blood proximal to the mixing site was from the patient’s heart. In contrast, the majority of blood distal to the mixing site was from the VA-ECMO circuit. As residual cardiac output increased, the location of mixing moved further distal down the aorta.

Video 1Particle tracking of blood flow in VA-ECMO with varying levels of residual cardiac function. A hemodynamic simulation of blood flow in a patient on VA-ECMO. Particles were injected at the site of the VA-ECMO inlet (right iliac), and the aortic annulus. Particles injected at the aortic annulus were colored blue to represent relative hypoxia when compared to those injected at the right iliac. Residual cardiac function was increased from 0.0 to 2.0 L/min·m^2^ to demonstrate the range of residual function a person on VA-ECMO could potentially have (MP4 85728 kb).


### Relative Blood Flows Along Branch Points

In Fig. 2, it was demonstrated that mixing of the two sources of blood flow occurs in the arch of the aorta. This was further analyzed and the relative contribution of blood flow from each source was quantified in each major branching vessel. This is important as each major branch of the aorta supplies different organ systems that vary in their sensitivity to hypoxia. Analysis was based upon the blood supply to two major organs: the brain and the heart.

**Fig. 3:**
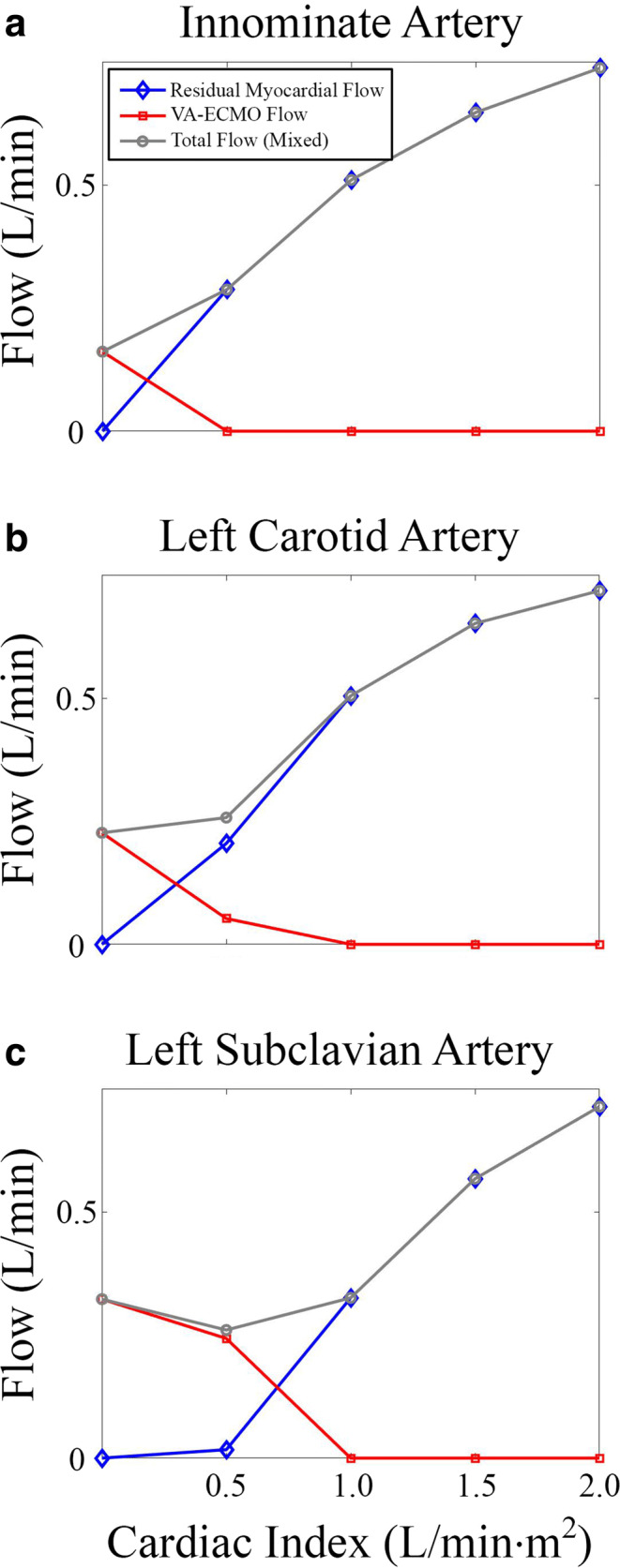
Blood flow analysis of major vessels supplying the brain. Volumetric flows of the innominate (**a**), left carotid (**b**), and left subclavian (**c**) arteries were analyzed at increasing residual cardiac indexes. Data shown are total blood flow through each vessel which was further broken down into flow from each source (residual cardiac function versus VA-ECMO)

Clinically, the major focus of NSS is cerebral oxygenation as even short periods of hypoxia can induce hypoxic brain injury/death, so initial analyses were on the major vessels that supply the brain. Figure 3 demonstrated the quantification of the total volumetric flow through the major vessels that contribute to cerebral perfusion: the innominate, left carotid, and left subclavian arteries. In each branching vessel, analyses quantified total volumetric flow which was split based upon the inlet source of that blood (i.e., whether it was from the residual cardiac output or VA-ECMO). With a residual cardiac index of “0,” unsurprisingly, all blood flow at each of the three outlets was from the VA-ECMO circuit. With a residual cardiac index of 0.5 L/min·m^2^ (representative of severe cardiogenic shock), overall flow to the innominate and left carotid slightly increased while overall flow to the left subclavian slightly decreased. More than 2/3 of blood flow through the innominate and left carotid originated from the patient’s residual cardiac function, whereas all of the blood flow through the left subclavian originated from the VA-ECMO circuit.

When the residual cardiac index was increased to 1.0 L/min·m^2^ (representative of moderate cardiogenic shock), total flow through all of these vessels increased slightly. Nearly all blood flow through the innominate and left carotid originated from the patient’s residual cardiac function, whereas in the left subclavian, there were roughly equal amounts of blood flow from each origin. When the residual cardiac index was increased to 1.5 L/min·m^2^ (representative of mild cardiogenic shock), again total flow in each vessel increased. At this level of residual cardiac function, all flow from the VA-ECMO circuit was displaced by residual cardiac output resulting in no cerebral perfusion from the VA-ECMO circuit.

It should be noted that the final residual cardiac index of 2.0 L/min·m^2^ would not exist clinically as this level of myocardial function would not require VA-ECMO but was simulated for completion. At this level, a similar trend was observed. While a patient would unlikely be on VA-ECMO even with a cardiac index of 1.5 L/min·m^2^; it is important to note that with a myocardial index representative of moderate/severe cardiogenic shock range (1.0 L/min·m^2^), most of the blood flow through the vessels that perfused the brain originated from the patient’s native residual heart function. Finally, the mixture of blood within each branching vessel appeared to be directly related to each vessel’s distance from the aortic annulus. For example, at each residual cardiac output, the fractional distribution of flow was similar in the innominate and left carotid, whereas the left subclavian required double the residual cardiac output to displace all VA-ECMO flow when compared to the innominate.

**Fig. 4:**
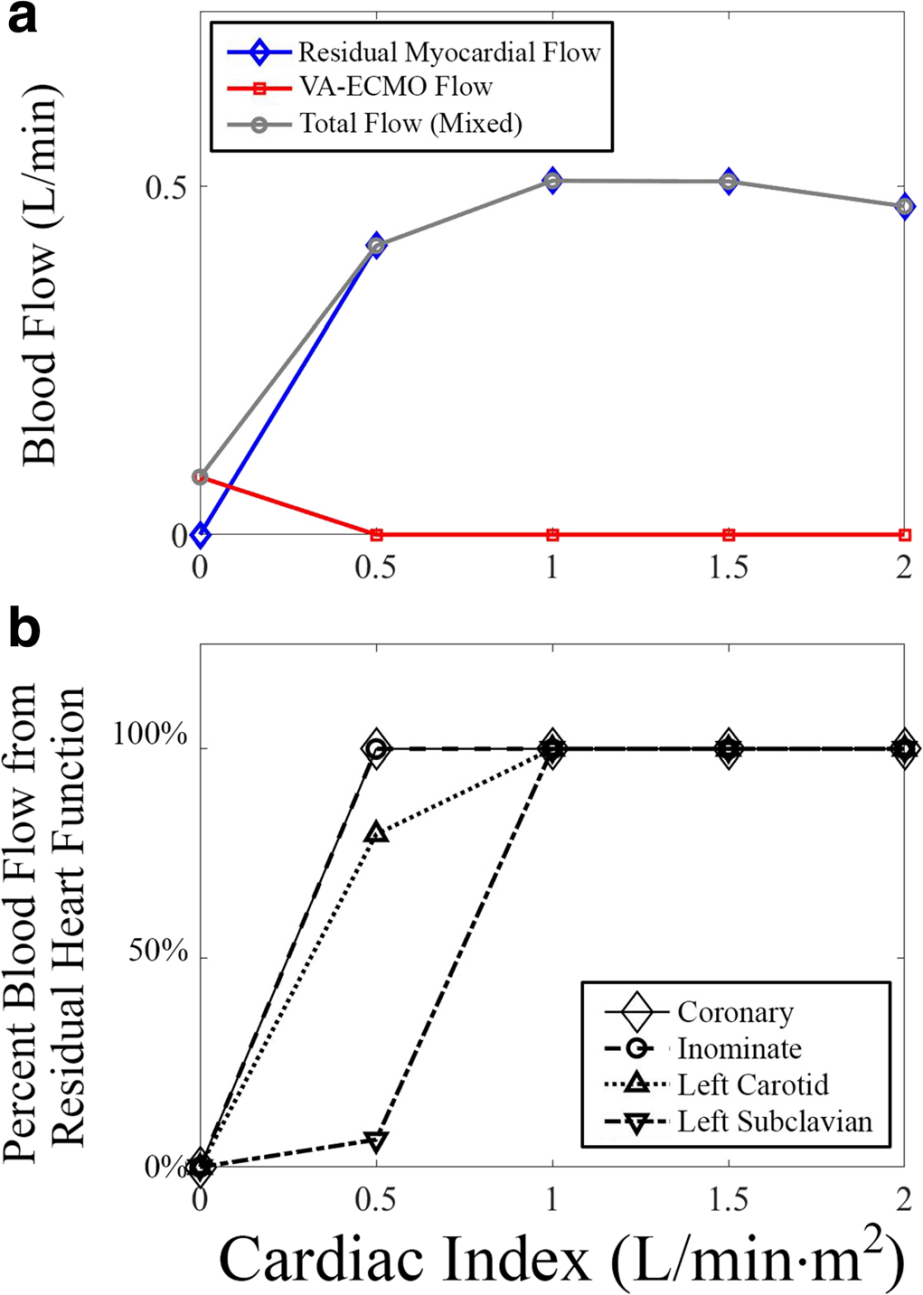
Blood flow analysis of coronary arteries. **a** Volumetric flow of the coronary arteries was analyzed at increasing residual cardiac indexes. Data shown are total blood flow through each vessel which was further broken down into flow from each source (residual cardiac function versus VA-ECMO). **b** Blood supply to the heart (coronaries) was compared with the brain (innominate, left carotid, left subclavian) as a percent of total flow from residual cardiac function

Next, myocardial perfusion was analyzed by quantifying blood flow through the coronary arteries (Fig. 4a). At a cardiac index of 0.0 L/min·m^2^, all blood flow through the coronary arteries originated from the VA-ECMO circuit. As soon as the residual cardiac index was increased to 0.5 L/min·m^2^ and above, all blood flow through the coronary arteries originated from the patient’s residual cardiac function. This remained the case for the rest of the simulations. Likely this is due to the close proximity of the coronary arteries to the aortic annulus.

To compare perfusion of the heart with the brain, the percent of blood flow that originated from the patient’s residual cardiac function in each branching vessel (coronaries, innominate, left carotid, and left subclavian) was plotted versus residual cardiac function (Fig. 4b), and it was found that the percent of blood flow from the residual cardiac function increased more slowly the more distal the branching vessel was from the aorta. These results are significant because they demonstrate that even though the brain is the most sensitive organ to hypoxia, the heart is the most likely to be affected by NSS.

**Fig. 5:**
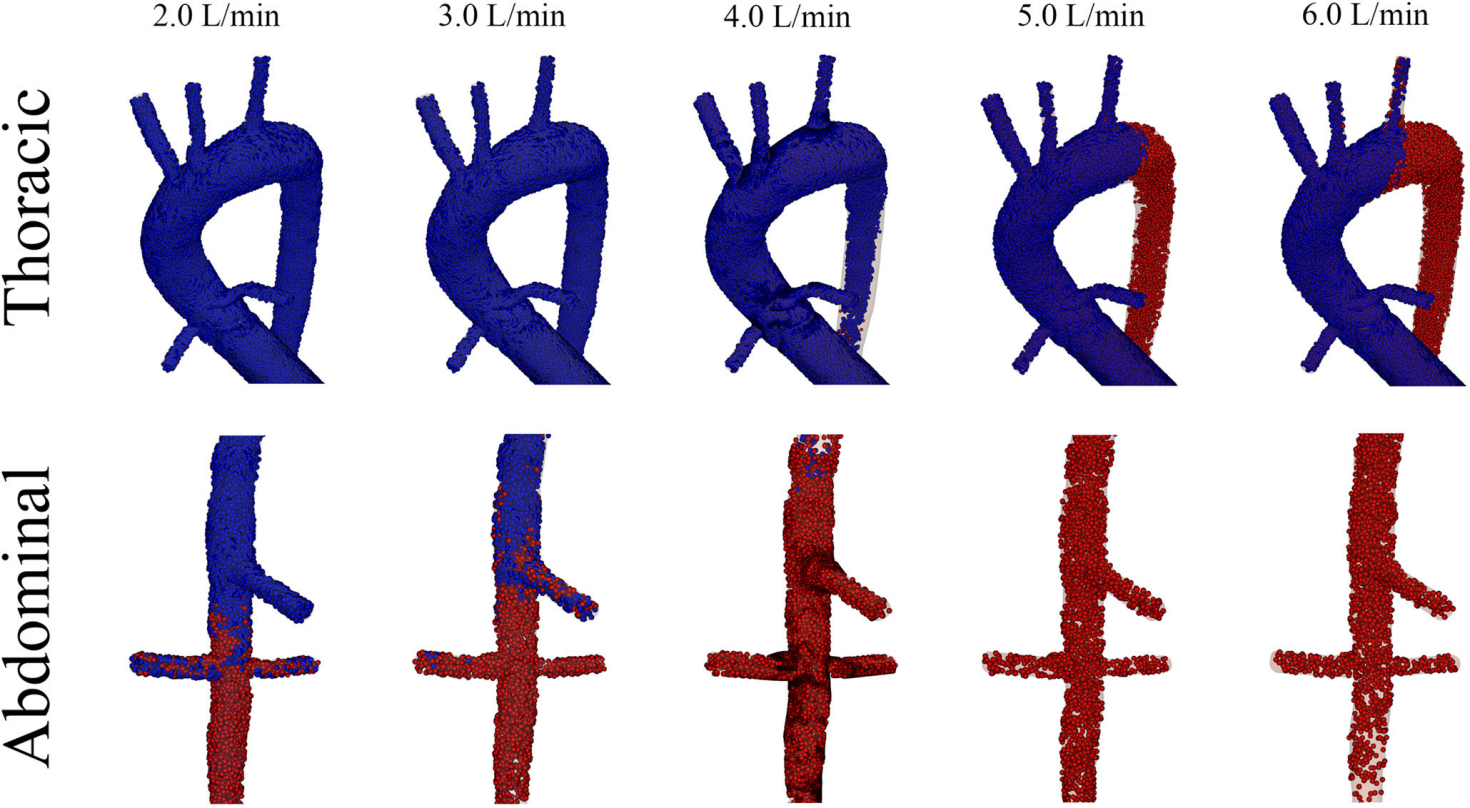
Mixing site as function of VA-ECMO flow. The site at which the two sources of blood mix was visualized after the simulations reached steady state. As the VA-ECMO flow was increased, the site of mixing shifted proximal, starting in the abdominal aorta (VA-ECMO flow of 2.0 L/min) reaching the thoracic aorta (VA-ECMO flow of 6.0 L/min)

When NSS is developing, many physicians try to improve oxygen delivery by increasing flow from the VA-ECMO circuit thereby shifting the site of mixing more proximally along the aorta. To test the hypothesis that increasing VA-ECMO flow shifts blood flow more proximal, cardiac index was held constant at 1.0 L/min·m^2^ and VA-ECMO flow was varied from 2.0 to 6.0 L/min. At relatively low VA-ECMO flows, mixing was observed in the abdominal aorta. As flow was increased, the mixing was observed more proximally in the arch of the thoracic aorta (Fig. 5).

### Clinical Evidence of Myocardial Injury Due to NSS-Induced Hypoxia

**Fig. 6:**
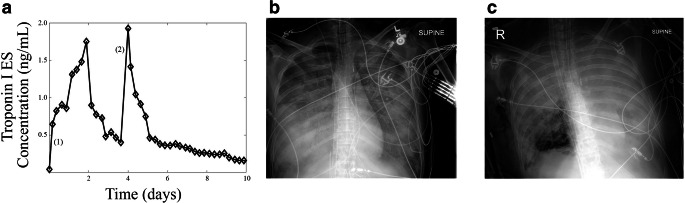
Myocardial injury in patient with north-south syndrome. A patient with distributive shock secondary to sepsis from bacterial pneumonia suffered cardiac arrest and prolonged resuscitation. Serum troponin (**a**) was plotted as a function of time. Troponin was tracked every 6 h. Patient was placed on VA-ECMO for sepsis-induced cardiomyopathy at (1). Myocardial recovery progressed and patient developed NSS at (2). Chest X-ray of patient at hospital admission (**b**) and after development of NSS (**c**) shows significant lung injury

Evidence of myocardial injury secondary to NSS-induced hypoxia has not been described in the literature, likely due to the difficulty of measurement. After performing a chart review of all the patients who were placed on VA-ECMO for acute cardiogenic shock at the University of Minnesota between 2015 and 2019, one patient was identified who had concomitant ARDS and regular cardiac-specific troponin measurements (a serum marker of myocardial necrosis) after being placed on VA-ECMO (Fig. 6).

This patient was admitted to the ICU with distributive shock secondary to sepsis from streptococcus pneumonia. Several hours after admission, the patient suffered a pulseless electrical activity cardiac arrest with prolonged resuscitation and subsequently went into cardiogenic shock (post-arrest ejection fraction of < 10%) and was placed on VA-ECMO. The new cardiomyopathy was thought to be secondary to stress from pneumonia/resuscitation. This patient had no evidence of coronary artery disease. Troponin values were measured every 6 h and initially were thought to be elevated secondary to cardiac arrest and resuscitation. This was supported by a decrease in serum troponin after the initiation of VA-ECMO. However, towards the end of day 3, the patient demonstrated improvement in cardiac function (ejection fraction of 40%) and became acutely hypoxic. Until that point, the patient was adequately supported with 3.6 L/min of flow from the VA-ECMO source.

Several hours prior to improvement of cardiac function, a VA-ECMO turn-down study was conducted. When VA-ECMO flow was turned down to 2.0 L/min, arterial blood gas oxygenation from the right radial artery demonstrated a PO2 of 29 mmHg. This measurement was done approximately 6 h before the 2nd troponin peak in Fig. 6. The oxygen level normalized returning the flow back to 3.6 L/min. Over the next 3 h, oxygenation as measured from the right radial worsened. A PO2 of 51 mmHg prompted increasing the flow to 5.5 L/min which temporarily resolved the hypoxia. Worsening hypoxia via pulse oximetry prompted conversion from VA-ECMO to VAV-ECMO by adding an additional cannula providing oxygenated blood to the pulmonary artery. In this configuration, blood flowing through the lungs would be pre-oxygenated, resulting in blood from residual cardiac function being oxygenated. As demonstrated by Figs. 3 and 4, hypoxia in the innominate artery would also be consistent with hypoxia in the coronaries. As this analysis was retrospective, only association can be determined. In this case, the patients’ second troponin peak, which trended down after the ECMO reconfiguration, is consistent with hypoxic injury from NSS.

## Discussion

In this study, we describe a quantitative investigation of the hemodynamics in a patient on VA-ECMO. More specifically, a mathematical model was developed that described the original source of blood flow (residual cardiac output from an acutely failing heart versus VA-ECMO) at each major aorta branch point. The mathematical model developed was used to test the following hypothesis: Residual cardiac function in a patient on VA-ECMO with concomitant lung injury will result in end-organ (cerebral or myocardial) hypoxia. Simulations supported that NSS could account for hypoxic injury to the brain and/or heart.

### Identification of Precipitating Conditions

NSS will only develop if there is significant lung injury and enough residual cardiac output to displace blood flow from the VA-ECMO device. It must be noted that most patients with ARDS can be supported using a ventilator alone, so for NSS to develop, the lung injury must be advanced enough that respiratory support requires external oxygenation. In our simulations, a residual cardiac index of 1.0 L/min·m^2^ was required to displace VA-ECMO flow to the brain. While there is no routine clinical way to estimate cardiac output or pulmonary oxygenation in a patient on VA-ECMO, our results would suggest that there is value to taking a daily chest radiograph (to assess lung injury) and limited transthoracic echocardiogram (to assess systolic function). Performing these measurements would help physicians identify patients who are at risk of developing NSS. To avoid cerebral hypoxia in patients with significant lung injury, the intensive care team should be prepared to reconfigure the VA-ECMO circuit to VAV or VV-ECMO when there is mild recovery of cardiac function. While recognizing that these conditions (severe lung injury and myocardial recovery) lead to cerebral hypoxia from NSS, our study adds to the literature demonstrating that this can occur at a residual native cardiac index of less than 1.0 L/min·m^2^.

### Quantification of NSS and Cerebral Hypoxia

The clinical problem with NSS is that it can rapidly induce hypoxic injury to the brain. Immediate recognition of NSS is critical as treatment requires upgrading VA-ECMO to VAV-ECMO. This requires placement of an additional cannula in the pulmonary artery that can supply the lungs with oxygenated blood. When a clinician is observing hypoxia-related complications in a patient on VA-ECMO, and it is suspected to be secondary to NSS, he/she should keep in mind where the anatomical site of mixing is likely located. While making arrangements to reconfigure the ECMO circuit, a physician can temporize the situation by shifting the location of mixing more proximally in the aorta. This can be done by increasing the flow from the VA-ECMO pump (Fig. 5) or administering a negative-inotropic agent to reduce the native residual cardiac index (Fig. 2).

### Myocardial Recovery in NSS

Simulations from our studies have shown that myocardial hypoxia has the potential to develop with significantly less residual cardiac function than required to induce cerebral hypoxia. While the literature has focused on cerebral hypoxia, no studies have investigated myocardial hypoxia. This is likely not discussed for two reasons. First, there are no routine clinical ways to directly measure myocardial oxygen saturation. Secondly, patients who are on VA-ECMO are likely to be undergoing multi-system organ failure. In this setting, impaired myocardial recovery is much more likely to be explained as secondary to the underlying disease process than from hypoxia due to NSS.

Simulations in our studies demonstrate that generally, with increasing residual cardiac function or decreased VA-ECMO flow, the site of mixing moves distally along the aorta. Vessels proximal to the mixing site are generally supplied by residual cardiac function and those distal are supplied by VA-ECMO support. At relatively low levels of residual cardiac function (0.5 L/min·m^2^), the composition of blood that supplies the innominate artery is similar to that of the coronaries. This finding suggests that in most circumstances, monitoring of oxygenation through the patient’s right hand reflects that of myocardial oxygenation. The exception to this is likely at extremely low levels of residual cardiac function. If the residual cardiac function does not exceed the capacity of coronary blood flow, then all hypoxic blood will likely drain through the coronaries, resulting in a discrepancy between oxygen levels in the coronaries and innominate. Although not specifically tested in this study, we hypothesize this would likely occur at residual function of less than 0.2 L/min·m^2^. However, this finding is not generalizable as the actual limit will likely vary significantly depending upon each patients’ anatomy. This result highlights the importance of optimizing ventilator therapy for all patients on VA-ECMO, especially in those with extremely low levels of cardiac function.

### Severe COVID-19 Infection Predisposes Patients to Development of NSS

The Extracorporeal Life Support Organization (ELSO) has been tracking patients who were placed on ECMO for infection with the COVID-19/SARS-CoV-2 virus [19]. These data have not been formally published, but are publicly available on their website (www.elso.org). Within this registry, 3817 patients have been placed on ECMO, with a 55% rate of patients surviving until hospital discharge. In this registry, most patients required VV-ECMO with 4% of patients required VA-ECMO. While survival numbers are not yet published for those on VA-ECMO, it is likely that survival will be worse than the average of the cohort, as acute cardiogenic shock in these patients is associated with multi-system organ failure. Patients on VA-ECMO due to COVID-19 represent a population at high risk of developing NSS, which we have also observed at our own institution. Early recognition of NSS and appropriate optimization of VA-ECMO treatment (conversion to VAV or VV-ECMO) on this population to avoid NSS-related hypoxia will likely result in decreased mortality.

### Hemodynamic Quantification of Blood Flow in Patient’s on Peripheral VA-ECMO

Mixing of competing blood sources in mechanical circulatory support is a topic that has been studied for nearly 20 years. One of the first studies by Kar et al. was a 2D simulation with approximately 7000 elements that identified regions of stagnation in the aorta in a patient with an implanted ventricular assist device [20]. While this study was state-of-the-art at the time, modern simulations include several million elements in 3D. There are three key contemporary mathematical studies that have quantified hemodynamics in VA-ECMO-supported patients. The first was that by Stevens et al. which determined where in the aorta that the mixing site was located [5]. The variables analyzed in this study were stroke volume and VA-ECMO output. Within clinically realistic ranges, the stroke volume was varied, and the site of mixing in this study was found to be at different locations within the aortic arch, a result consistent with our studies. The second study was a follow-up by the same group, where the relative oxygenation of the great vessels was estimated under a variety of different physiologically relevant flows. Analyses presented demonstrated that lower LV oxygenation resulted in more extreme hypoxia to the cerebral vessels [21]. The final study is by Feiger et al. where adjustment of the ECMO cannula site predicted changes in blood flow over a 1D model of the cardiovascular system [22]. Sites analyzed were axillary, central, and two femoral configurations. It was found that the closer the cannula was to the coronary arteries, the less likely NSS was to develop, a finding that is consistent with our studies.

Our study improved upon the previous for several reasons: First, our simulations were the first to examine blood flow to the heart as previous models did not include coronary arteries, but rather the major focus was cerebral perfusion. Second, residual cardiac function was quantified as a cardiac index, so direct clinical correlations were able to be drawn. While intermittent opening of aortic valve will likely occur at low levels of cardiac function, the average behavior will likely be similar to those presented in Figs. 2 and 5. Finally, geometries were derived from a high-resolution CT scan performed on a cadaver, which eliminated traditional limits on radiation and contrast dosages, resulting in a high-resolution 3D geometry.

### Limitations

Our study has several weaknesses. The first is the parameterization of the boundary conditions. Flows at each branching vessel were approximated as a 3rd-order Windkessel model (resistance and capacitance estimates for each branch) and were parameterized from flow data in the 2013 study by Stefanov et al. where MRI was used to quantify flows in the aortic branches [13]. The major weakness is that it reflects the distribution of flow in that of a healthy human, rather than one on VA-ECMO. While changes in coronary flow have not been directly measured in a patient on VA-ECMO, many have hypothesized that hemodynamic changes associated with VA-ECMO result in decreased coronary flow, which has been offered as one of the several mechanisms of increased short-term mortality associated with VA-ECMO [23]. Using a lumped parameter model, Donker et al. estimated that loading conditions could change coronary flow about 25% [24]. Likely, patient variability would have a similar magnitude of effect. While this is a significant difference in flow, changes to the distribution of blood flow in our model likely will only result in small changes of the mixing site location, the main focus of this study. The general conclusions about the hypotheses will likely remain the same.

The second is that the output of the model was a percent of blood flow that originated from the residual cardiac function instead of a percent oxygen saturation. This was necessary because there currently exists no routine clinical way to measure oxygen saturation of residual cardiac output, so any level of oxygenation would be arbitrary. Instead of assigning an oxygen level, we focused on tracking the pathway of blood flow from the aortic annulus and identifying which vessels it perfused, as this source of blood is the one causing hypoxia in NSS. From a portable chest radiograph, an experienced intensivist can determine if there is enough lung injury to induce significant hypoxia.

In summary, NSS is a rare but serious complication of VA-ECMO. Most importantly, physicians should be aware of the conditions under which NSS develops as early recognition can give the intensive care team valuable time to arrange for reconfiguration of the VA-ECMO circuit.

## Supplementary Information

ESM 2 (DOCX 63 kb).

## Declarations

### Animal Studies

No animal experiments were carried out for this study.
