# Supplementary material for: Concomitant Respiratory Failure Can Impair Myocardial Oxygenation in Patients with Acute Cardiogenic Shock Supported by VA-ECMO
Source: J Cardiovasc Transl Res. 2021 Feb 23;15(2):217–26. doi: 10.1007/s12265-021-10110-2 (PMC7901681; doi:10.1007/s12265-021-10110-2)
Supplement: Supplementary file 2 — (DOCX 63 kb). [file 12265_2021_10110_MOESM2_ESM.docx]

Supplemental Data for Manuscript: Concomitant Respiratory Failure Can Impair Myocardial Oxygenation in Patients with Acute Cardiogenic Shock Supported by VA-ECMO

*3-Element Windkessel Boundary Conditions.* Boundary conditions to the CFD model were set up to approximate a physiological distribution of blood flow by approximating the pressure at each outlet using a 3-Element Windkessel model. This technique is consistent with methodology described in previous studies, both by those from our group and others.^1-3^ Assuming that the arterial pressure can be separated into reservoir and wave components, the reservoir pressure was estimated following the method described by Aguado-Sierra et al given an approximate pressure waveform measured in a recovering VA-ECMO patient.^3^


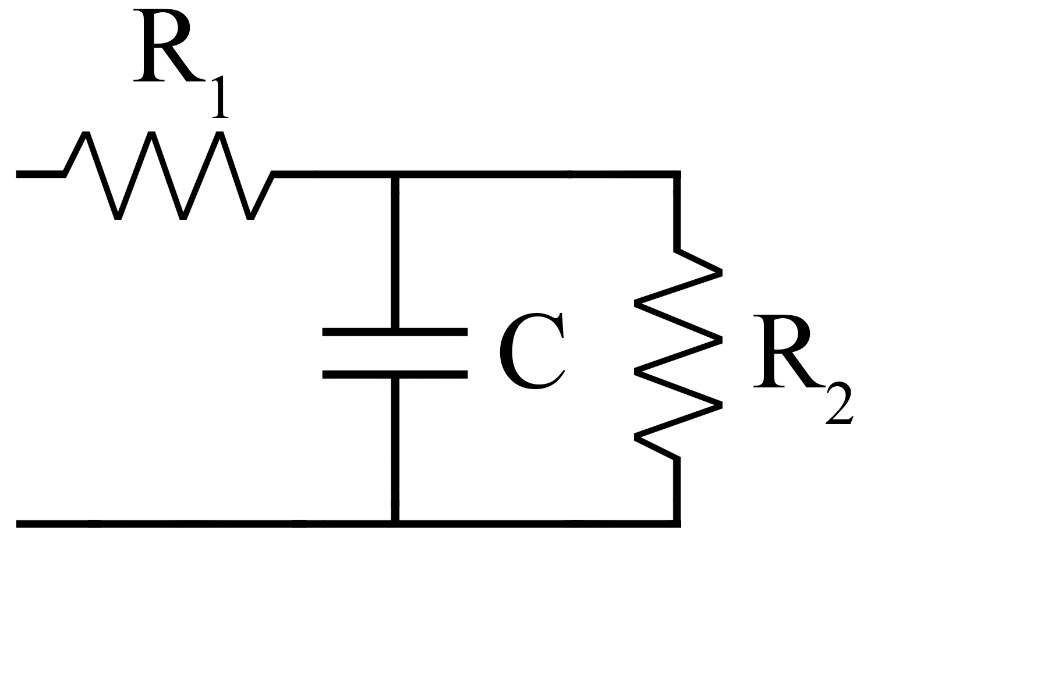


Figure 1: Circuit Diagram of 3-Element Windkessel Model

**Figure 1** is an illustration of 3-Element Windkessel Model as a circuit diagram, where there are two resistive elements (R_1_, R_2_) and one capacitive element (C). From the estimation of the reservoir pressure, two constants are obtained: b = 1/τ and P_∞_ (pressure at which flow is zero). Furthermore, the time constant “τ,” which has the relationship of τ = RC, which refers to the resistance (R) and capacitance (C) of the components in parallel in the 3-Element Windkessel configuration. Provided that in the baseline condition of our CFD simulation, the inflow to the aorta is known, we can then estimate the R and C values by solving:

$$C=\frac{e^{\frac{-t}{\tau}}\int_{0}^{t} Q(t)e^{\frac{t}{\tau}}dt}{P(t)-P_{\infty}e^{\frac{t}{\tau}}}$$

In the above equation, Q(t) is the inflow flow curve at the baseline condition (without VA-ECMO) and P(t) is the measured pressure waveform. Once the Capacitance (C) is calculated, then the Resistance is obtained using the following relationship:

$$R=\frac{1}{bC}$$

These values were used as an initial approximation to the boundary conditions to the CFD model. The third component of the windkessel model was iteratively estimated by approximating the normal outflow at each aortic branch. The total resistance was adjusted to provide an approximately normal pulse pressure by constraining their ratio (R1 = 4 R2). To further decrease the flow in the coronaries to approximate a normal flow through them, the Capacitance C was reduced by 0.0001, while it was increased in the iliac arteries to maintain a physiological distribution of blood flow. Flow through each branching vessel was consistent with previous studies.^4^ The final values obtained are outlined in **Table 1**.

Table 1: 3-Element Windkessel Coefficients used for Boundary Conditions of Branching Vessels

| Branch | R1 (dyne**^.^**sec/cm^5^) | R2 (dyne**^.^**sec/cm^5^) | C (cm^5^/dyne) |
| --- | --- | --- | --- |
| Superior Mesenteric | 2183.9 | 8735.4 | 0.000248 |
| Left Renal | 3562.4 | 14249.6 | 0.000248 |
| Right Renal | 3722.4 | 14889.6 | 0.000248 |
| Left Subclavian | 5342.4 | 20965.6 | 0.000248 |
| Left Carotid | 5242.4 | 20969.6 | 0.000248 |
| Innominate | 5322.4 | 21289.6 | 0.000248 |
| Left Coronary | 15444.8 | 61779.2 | 0.000148 |
| Right Coronary | 15444.8 | 61779.2 | 0.000148 |
| Left Iliac | 550.6 | 2202.4 | 0.000348 |

References:

1. Aliseda A, Chivukula VK, Mcgah P, Prisco AR, Beckman JA, Garcia GJ, Mokadam NA and Mahr C. LVAD outflow graft angle and thrombosis risk. *ASAIO journal (American Society for Artificial Internal Organs: 1992)*. 2017;63:14.

2. Mahr C, Chivukula V, McGah P, Prisco AR, Beckman JA, Mokadam NA and Aliseda A. Intermittent aortic valve opening and risk of thrombosis in VAD patients. *ASAIO journal (American Society for Artificial Internal Organs: 1992)*. 2017;63:425.

3. Aguado-Sierra J, Alastruey J, Wang J, Hadjiloizou N, Davies J and Parker K. Separation of the reservoir and wave pressure and velocity from measurements at an arbitrary location in arteries. *Proceedings of the Institution of Mechanical Engineers, Part H: Journal of Engineering in Medicine*. 2008;222:403-416.

4. Stefanov F, McGloughlin T, Delassus P and Morris L. Hemodynamic variations due to spiral blood flow through four patient‐specific bifurcated stent graft configurations for the treatment of abdominal aortic aneurysms. *International journal for numerical methods in biomedical engineering*. 2013;29:179-196.
